# A CNN‐Based Deep Learning Architecture for Discriminating Botanical Adulteration and Complexities Among Commercial Apiaceae Medicinal Species

**DOI:** 10.1002/fsn3.71634

**Published:** 2026-03-15

**Authors:** Elyas Aryakia, Ersam Aryakia

**Affiliations:** ^1^ Plant Bank Iranian Biological Resource Center (IBRC), ACECR Tehran Iran; ^2^ Information Technology Group Payam Noor University of Sabzevar Sabzevar Iran

**Keywords:** adulteration, Apiaceae, CNNs, confusion matrix, t‐SNE visualization

## Abstract

This study presents a CNN‐based deep learning framework to automate the accurate authentication of mericarp seeds from 15 commercially important medicinal Apiaceae species, which exhibit subtle morphological overlaps and face risks of toxic adulteration (e.g., hemlock contamination), threatening consumer safety and trade integrity. Six CNN models were evaluated, with DenseNet121 demonstrating superior performance in terms of accuracy, precision, recall, F1‐score, and convergence stability, followed by MobileNetV2, InceptionV3, and VGG16. Confusion matrix analysis revealed that higher‐resolution inputs significantly improved discrimination, particularly for similar seeds like *Anethum graveolens* L. and *Apium graveolens* L. Batch size minimally influenced outcomes. Analysis of accuracy‐loss dynamics further indicated that EfficientNetB0 and ResNet50 underperformed, while DenseNet121, which excelled in performance, convergence stability, strong generalization, and minimal overfitting, highlighted the critical role of architectural design in feature learning and optimization of key performance metrics. Additionally, t‐SNE visualization confirmed DenseNet121's superior feature learning, achieving distinct separation of all 15 species and resolving intricate visual ambiguities that confound traditional methods and limit clearer clustering in other architectures. These findings underscore the potential of CNNs as scalable tools for botanical authentication—particularly for detecting adulteration and species complexities using seed digital morphometric characters—to safeguard public health and reinforce supply‐chain safety.

## Graphical abstract

This study presents a deep learning framework for the automated authentication of 15 medicinal Apiaceae species using digital images of their mericarps. The pipeline integrates an augmentation module—incorporating rescaling, rotation, and flipping—to improve model generalization, followed by feature extraction using pre‐trained DenseNet121 as the core architecture. A classification head comprising global average pooling, dropout, and dense layers refines the learned representations for species‐level discrimination. Model performance is monitored via training and validation accuracy curves, confirming robust differentiation of morphologically similar seeds, including commercially important taxa such as cumin, fennel, and coriander. The proposed approach offers a scalable and non‐destructive tool for quality control in botanical supply chains.

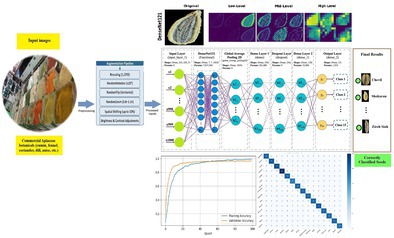

## 1 Introduction

Modern agriculture and the related food‐medicinal supply chain are undergoing a profound digital transformation. The integration of artificial intelligence (AI) and machine learning (ML) is no longer a futuristic concept but a practical necessity for achieving higher efficiency, traceability, and sustainability across the value chain (Aryakia et al. 2017). Deep learning—particularly Convolutional Neural Networks (CNNs)—has emerged as a central enabler of this shift by facilitating precise, automated analysis of complex visual data captured from drones, satellites, smartphones, and in‐line quality‐control cameras (Attri et al. 2023; El Sakka et al. 2025). Unlike classical statistical models, CNNs autonomously extract multiscale features, thereby eliminating human bias and subjectivity and allowing real‐time decision‐making. Applications already encompass early pest and disease detection, soil and canopy monitoring, and yield estimation, collectively reducing chemical inputs, curbing crop losses, and lowering the environmental footprint of farming operations (Mohanty et al. 2016; Koirala et al. 2019).

Beyond the field, CNNs have recently emerged as pivotal tools in seed industry, particularly for seed‐based detection technologies. Recent literature substantiates the efficacy of these models in classifying various plant varieties with high precision. For instance, advanced architectures like hybrid CNN‐Transformers and attention‐based models have demonstrated exceptional performance in rice classification, achieving accuracies exceeding 99% (Zheng et al. 2025; Prova 2025). Similarly, diverse CNN implementations—ranging from 1D models for mustard to 3D networks for corn—have proven successful in trait analysis and vigor evaluation, with some reaching 100% accuracy (Slebioda and Zawieja 2025; Wongchaisuwat et al. 2025). Furthermore, models such as EfficientNet‐B0 have been validated for assessing soybean damage and quality, confirming their practical utility (Chauhan et al. 2025; Dioses et al. 2025). These achievements underscore the untapped potential of image‐based deep learning for high‐throughput, non‐destructive authentication of economically important botanical commodities in industrial settings.

Another compelling reason for employing CNNs to identify and authenticate plant materials—particularly seeds—is the multitude of challenges inherent in classical botanical methods: they are labor‐intensive, require specialized expertise, and often falter when confronted with minute, morphologically similar taxa (Naghavi et al. 2019; Aryakia and Karimi 2018). These challenges are particularly pronounced in the Apiaceae family, where significant morphological overlap necessitates the use of complex phytochemical and molecular techniques for accurate taxonomy (Plunkett et al. 2018; Aryakia 2020). For instance, genera within the Apieae tribe, such as Anethum and Seseli, exhibit heterogeneity that morphological traits alone cannot resolve, often requiring molecular analyses (e.g., ITS, rps16) to clarify phylogenetic relationships (Jimenez‐Mejias and Vargas 2015). Similarly, distinguishing *Bunium persicum* from *Cuminum cyminum* within this family frequently demands DNA barcoding alongside phenotypic examination (Bansal et al. 2018). Moreover, recent studies demonstrate the efficacy of deep learning in quantifying subtle pollen morphology to infer ancient climatic adaptations in *Podocarpus* and in accurately classifying Western European flora at genus and family levels based on shared visual traits. These findings underscore deep learning's promising future in unraveling evolutionary histories and refining plant classification (Adaimé et al. 2025; Seeland et al. 2019). Consequently, integrating advanced computational technologies like CNNs offers a crucial, efficient complement to classical methods for overcoming these taxonomic complexities.

The consequences of misidentification extend far beyond academic taxonomy. Accurate identification of medicinal plants is critical for ensuring therapeutic efficacy, consumer safety, and trade confidence, yet the sector faces significant challenges regarding misidentification and fraud (Aryakia 2026). This issue is particularly prominent in the Apiaceae family, which includes globally valuable species like cumin, fennel, and anise used in pharmaceutical and food industries (Sayed‐Ahmad et al. 2017; Thiviya et al. 2022). Unfortunately, the high economic value and diverse applications of these species create incentives for intentional or unintentional substitution, often leading to the replacement of authentic plants with inferior or even harmful ones. A notable health risk arises from the morphological similarity between commercial *Foeniculum vulgare* and the poisonous *Conium maculatum* (hemlock), which contains potent neurotoxins and has caused poisoning incidents (Colombo et al. 2009). Beyond safety concerns, economic adulteration is frequent; for instance, expensive black cumin seeds (*Bunium persicum*) are often mixed with common cumin ( *Cuminum cyminum* ), while *Foeniculum vulgare* is frequently adulterated with *Anethum graveolens* , *Pimpinella anisum* , or *Cuminum cyminum* to reduce production costs (Ma et al. 2015; Bansal et al. 2018; Welke et al. 2015; Bisht et al. 2014). Such practices compromise food safety, consumer health, and the integrity of international trade, emphasizing the urgent need for rapid, reliable, and scalable authentication technologies. To address these critical issues, deep learning models, particularly Convolutional Neural Networks (CNNs), have emerged as powerful tools for detecting food fraud across various spices. For instance, CNNs have successfully identified walnut powder adulteration in cinnamon with 92.80% accuracy and distinguished genuine saffron from fake samples with 99.5% accuracy. Similarly, optimized DenseNet architectures achieved 98.92% accuracy in detecting adulterated red chili powder (Ku et al. 2023; Husaini et al. 2022; Brar et al. 2025). These results highlight the capability of deep learning to provide rapid, high‐precision authentication for ensuring food safety.

Considering the challenges of identifying and discriminating botanical complexities and adulterations in commercial medicinal Apiaceae seeds—particularly due to their small size and significant morphological overlap—this study introduces an end‐to‐end CNN‐based framework explicitly designed for food‐medicinal seed authentication and adulteration deterrence. We evaluate six state‐of‐the‐art architectures—VGG16, EfficientNet‐B0, MobileNetV2, DenseNet121, ResNet50, and InceptionV3—using high‐resolution images of mericarp seeds from 15 economically important Apiaceae species. Through this analysis, our aim is to quantify their achievable detection accuracies, assess their feasibility for deployment on standard computational hardware, and demonstrate the tangible benefits of this approach for food safety monitoring. The present work therefore transcends purely technical contributions: it lays the groundwork for practical, scalable, and cost‐effective countermeasures against botanical adulteration, ultimately supporting regulatory compliance, safeguarding public health, and reinforcing global market confidence in high‐value medicinal and culinary herbs.

## 2 Material and Methods

### 2.1 Seed Samples and Image Preparation

**FIGURE 1:**
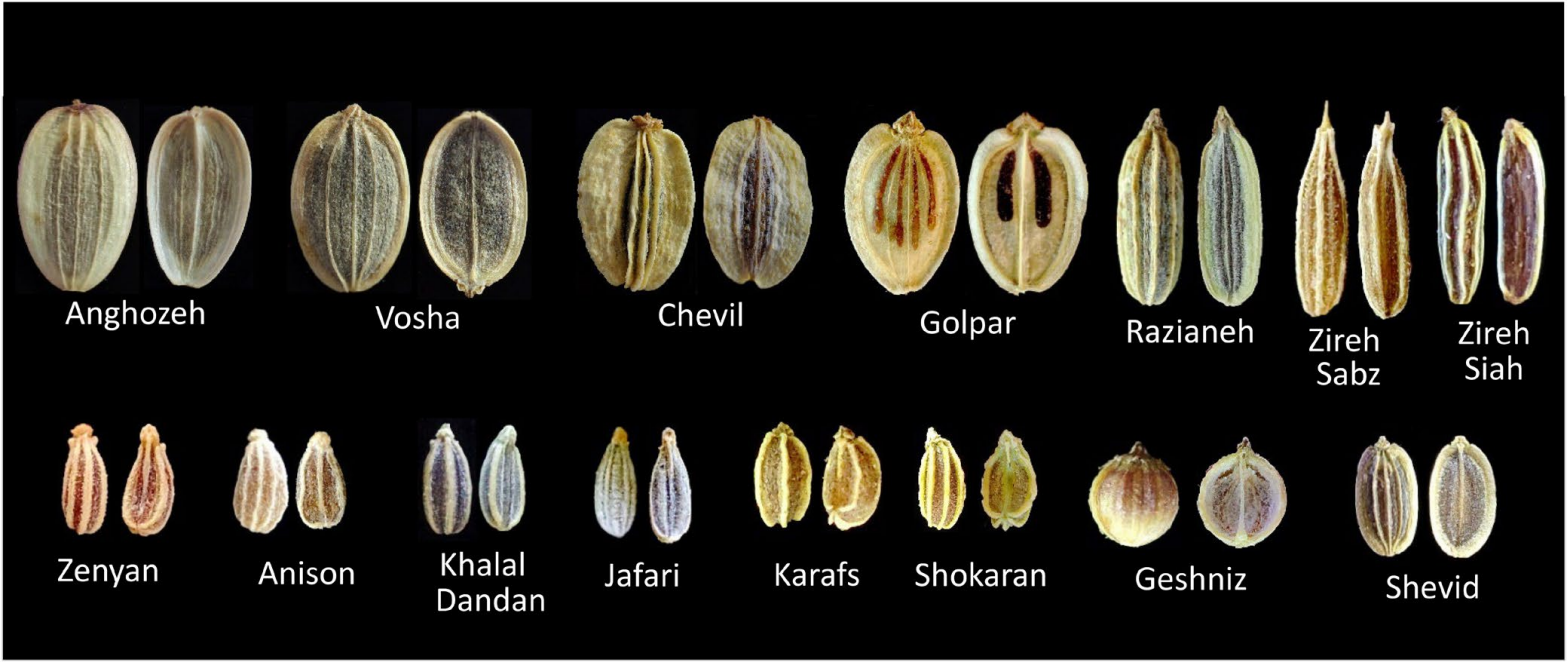
Dorsal and ventral views of seeds from 15 commercially important Apiaceae species included in this study.

The seed materials were prepared by the expert from the Iranian Biological Resource Center (IBRC), where this study was conducted. Their specifications are detailed in Table 1. To capture high‐quality images of the seed samples, a TELEPHOTO CAMERA equipped with SUPER MACRO MODE (optimized for close‐up imaging) was utilized, featuring a 5MP S5K5E9 1/5″ sensor with 1.12 μm pixels, a 50 mm f/2.4 lens, autofocus between 3 and 7 cm, and 2× optical zoom, as shown in Figure 1. A black background was employed to enhance contrast during the image preparation process. Images of each single seed were captured from both the adaxial and abaxial surfaces to ensure a comprehensive dataset. For each species, 200 images were prepared, totaling 3000 images. Images are saved in the memory in JPG format. Moreover, to improve robustness against variations in seed orientation, illumination, and minor appearance differences, all images were subjected to a unified data preprocessing and augmentation pipeline before being fed to the models. Pixel values were first rescaled to the [0, 1] range by dividing by 255, and then data augmentation was applied to synthetically increase intra‐class variability and reduce overfitting. The augmentation operations included random rotation (±20°), horizontal flipping, zooming (0.8×–1.2×), spatial shifting (up to 10%), and brightness/contrast adjustments. This pipeline was applied consistently to the training data of all CNN architectures to ensure a fair comparison and to isolate the impact of architectural differences rather than preprocessing discrepancies.

**TABLE 1:** Introduction and description of the plant species investigated in this study (Lucena et al. 2001; Duke 2002).

Persian name	English name	Scientific name	Seed shape	Seed size (mm)	Seed texture	Traditional use
Anghozeh	Asafoetida	*Ferula assa‐foetida* L.	Obovate or elliptical	12 × 7	Glabrous	Carminative, antispasmodic, expectorant, antimicrobial, mild antihypertensive
Anison	Anise	*Pimpinella anisum* L.	Oblong‐ovoid	3–5 × 2–2.5	Densely appressed setose‐hairy; vittae 2–4 in each furrow, 4–8 on commissure, nearly forming a continuous ring around seed. Seed face plane	Carminative, expectorant, galactagogue, mild estrogenic, antioxidant
Chevil	Ferulago	*Ferulago angulata* Boiss.	Elliptical	10–12 × 6	Glabrous or somewhat scabrous when young; wings 1.5–2.5 mm wide; dorsal ribs 8, commissural ribs 6	Potent antioxidant, anti‐inflammatory, neuroprotective, antimicrobial
Geshniz	Coriander	*Coriandrum sativum* L.	Hemispherical	2.0–4.5	Hemispherical mericarps, joined together, hardly separable at maturity, filiform ribs, slightly prominent, hard pericarp	Digestive aid, hypoglycemic, cholesterol‐lowering, anxiolytic, antimicrobial
Golpar	Giant Hogweed	*Heracleum persicum* Desf. ex Fisch., C.A.Mey. & Avé‐Lall.	Obovate, cuneate at the base	9–10 (−12) × 5–8	Apex emarginate, covered with minute aculeolate hairs or sometimes with filiform paleaceous hairs on the back, margin aculeolate	Carminative, diuretic, appetite stimulant, antioxidant, mild hypotensive
Jafari	Parsley	*Petroselinum crispum* (Mill.) Fuss	Narrowly elliptic	2–4 × 1.5–3	Fine longitudinal ridges	Diuretic, anti‐inflammatory, hepatoprotective, antioxidant, breath‐freshening
Karafs	Celery	*Apium graveolens* L.	Globulose	2–3 × 1.5–2	Minutely granulate surface	Antihypertensive, diuretic, anti‐inflammatory, sedative, antioxidant
Khalal Dandan	Bishop's Weed	*Ammi visnaga* (L.) Lam.	Falcate (sickle‐shaped)	2.2–5	5‐ribbed, with primary ribs broad and obtuse; pericarp slightly elevated above the vallecular vittae	Antispasmodic (ureter & bronchi), vasodilator, anti‐urolithic, photosensitizer (khellin)
Razianeh	Fennel	*Foeniculum vulgare* Mill.	Linear, nearly terete, ribbed	4–6 (−10) × 1.5–2.2 (−2.5)	Ribs marked carpophore divided to the base	Carminative, galactagogue, antispasmodic, estrogenic, antioxidant
Zire Sabz	Green Cumin	*Cuminum cyminum* L.	Slender and arcuate	5–7 × 1.6–2.8	Primary ribs short setulose, secondary ribs densely stellate setulose.	Carminative, digestive stimulant, hypoglycemic, anti‐inflammatory, antimicrobial
Shevid	Dill	*Anethum graveolens* L.	Compressed elliptic	3–5 × 2–2.5	Surface smooth, finely transversely striate	Carminative, digestive aid, lactation promoter, mild sedative, antimicrobial
Shokaran	Poison Hemlock	*Conium maculatum* L.	Elongated ovoid	2–4 × 1.5–2.5	With prominent, rugose, wavy, glabrous ribs, carpophore persistent, entire, sometimes bifid.	Historically antispasmodic & sedative; highly toxic—external analgesic use only with extreme caution
Zireh Siah	Caraway	*Carum carvi* L.	Oblong‐ellipsoid	3–5 × 1–2	Vittae 1 in each furrow, 2 on commissure.	Carminative, digestive aid, lactation promoter, antioxidant, mild diuretic
Vosha	Oshac, Gum Ammoniac	*Ferula ammoniacum* (D.Don) Spalik, M.Panahi, Piwczyński & Puchałka	Broadly elliptical	8 × 5–6	Brownish‐violet, initially white‐flocculose‐tomentose and later becoming glabrescent; with filiform, prominent juga; the vallecular area distinctly uni‐vittate; and the commissure 2–4‐vittate	Expectorant, antispasmodic, anti‐inflammatory, topical rubefacient
Zenyan	Ajowan caraways	*Trachyspermum ammi* Sprague	Ellipsoid with transverse grooves	1.2–2 × 1.2–1.8	Densely covered in whitish minute papillae	Carminative, antimicrobial, expectorant, bronchodilator, anthelmintic

### 2.2 Convolutional Neural Networks

**FIGURE 2:**
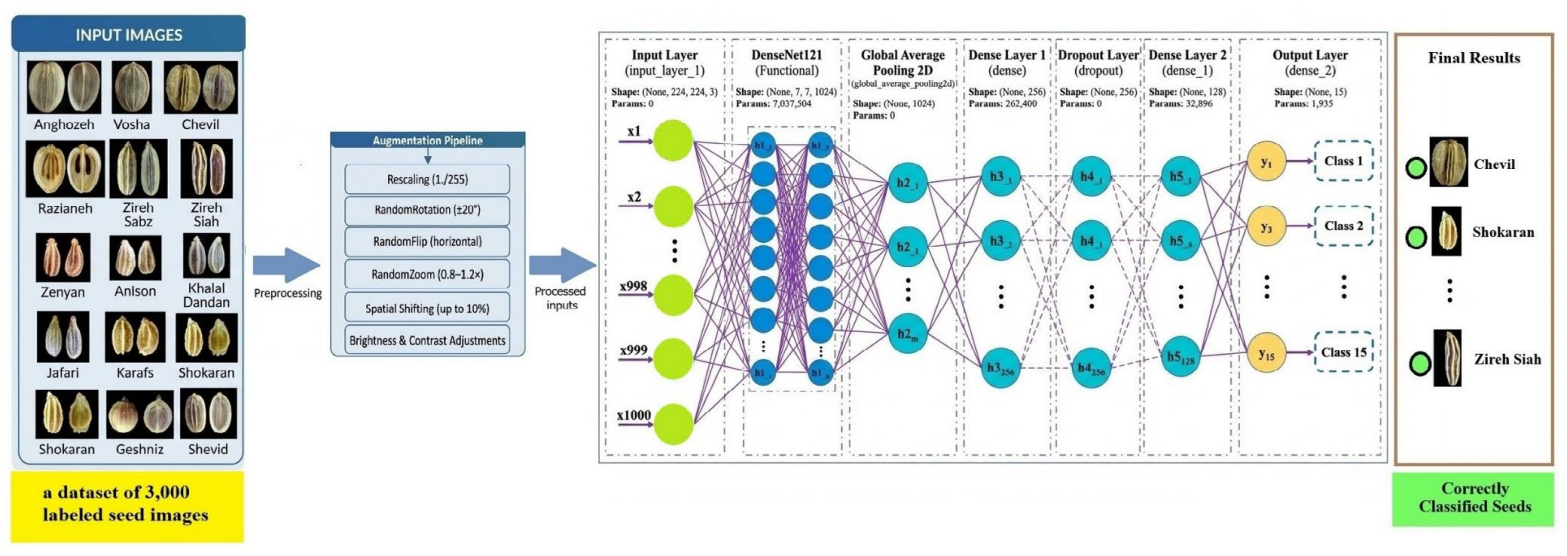
Overall pipeline and CNN architecture (illustrated for the DenseNet121 backbone). The augmentation pipeline and standardized classification head (Global Average Pooling + Dense 256 + Dropout 0.5 + Dense 128 + Softmax with 15 outputs) are used for all six backbones (EfficientNetB0, MobileNetV2, VGG16, ResNet50, DenseNet121, InceptionV3). Input size shown: 224 × 224 (other experiments with 64 × 64, 75 × 75, 299 × 299 are reported in the text).

This study employed six standard Convolutional Neural Networks (CNNs), including lightweight models—EfficientNetB0 and MobileNetV2—as well as heavyweight models—VGG16, ResNet50, DenseNet121, and InceptionV3—to evaluate and compare their performance in image classification. The characteristics of each of the models are mentioned in Table 2, and the schematic architecture of the CNN model is illustrated in Figure 2. The variables included image input sizes of 64 × 64 and 224 × 224 pixels (except for InceptionV3 with standard input sizes of 75 × 75 and 299 × 299), batch sizes of 32 and 64, and epoch counts of 50 and 100. A common transfer‐learning framework was employed for all networks: the ImageNet‐pretrained convolutional backbone (feature extraction layers) of each model was loaded and initially frozen to preserve generic low‐ and mid‐level visual features (edges, textures, basic shapes) learned from large‐scale natural images. On top of each backbone, we attached an identical classification head, consisting of a Global Average Pooling (GAP) layer, two fully connected layers with 256 and 128 neurons (each followed by a ReLU activation), a Dropout layer with a rate of 0.5, and a final Softmax output layer with 15 units corresponding to the 15 seed species. This standardized, regularized decision head (“enhanced features” in our implementation) was deliberately kept the same across all models to ensure that observed differences in accuracy, convergence speed, and generalization mainly reflect the intrinsic properties of the backbone architectures listed in Table 2. Training was performed using the Adam optimizer with a learning rate of 0.001, with early stopping (5‐epoch patience) to prevent overfitting based on validation loss. The dataset was split into 80% training and 20% validation sets, and a separate held‐out test set was used exclusively for final evaluation, ensuring that model selection and hyperparameter choices were guided only by training and validation performance. Performance was quantified using training and validation accuracy and loss, precision, recall, F1‐score, training time, and GPU resource consumption; by examining both endpoint metrics and full learning curves under this unified setup, we were able to directly relate each model's architectural characteristics (e.g., dense connectivity, inverted residuals, multi‐scale Inception modules) and the shared enhanced features of our implementation (transfer learning, common classification head, standardized augmentation) to its optimization dynamics and generalization behavior.

**TABLE 2:** Detailed characterization of CNN architectures utilized in the experimental framework (Yurdakul et al. 2024).

Model	Year introduced	Parameters	Input size	Depth	FLOPs (Giga)	Key architectural features	Typical use cases/strengths
VGG16	2014	138 M	224 × 224	16	15.5	Sequential 3 × 3 convolution blocks, largest parameter volume	Foundation for transfer learning in computer vision
EfficientNetB0	2019	5.3 M	224 × 224	237	0.39	Compound scaling (depth/width/resolution), MBConv with Squeeze‐and‐Excitation	Optimized for resource‐constrained edge devices (mobile, IoT)
MobileNetV2	2018	3.4 M	224 × 224	53	0.3	Inverted residual blocks, linear bottlenecks, reduced memory footprint	Real‐time processing in mobile applications
DenseNet121	2017	8 M	224 × 224	121	2.8	Dense connectivity (each layer connects to all subsequent layers), mitigates vanishing gradient	High‐detail image classification (e.g., medical disease detection)
ResNet50	2015	25.6 M	224 × 224	50	3.8	Residual blocks with skip connections, enables training of very deep networks	Transfer learning for diverse tasks (object, face, text recognition)
InceptionV3	2015	23.9 M	299 × 299	48	5.7	Factorized convolutions (7 × 7 → 1 × 7 + 7 × 1), auxiliary classifiers	High‐resolution image processing (e.g., satellite imagery analysis)

## 3 Result and Discussion

The results show that each model, depending on its architecture, number of parameters, and input configurations (e.g., image dimensions and batch size), exhibits distinct characteristics, consistent with findings reported in other studies (Laabassi et al. 2021; Yurdakul et al. 2024) (Table 3). Furthermore, the results of the correlation table generally corroborate these findings, given the significant relationships observed between the measured metrics for each model (Table 4). This table showed that performance metrics such as test accuracy, precision, recall, and F1‐score exhibit strong, positive, and significant correlations with each other (with coefficients ranging from 0.95 to 1.00). This demonstrates a high level of consistency among these metrics; for instance, an increase in test accuracy is accompanied by improvements in precision and recall. On the other hand, test loss shows a significant negative correlation with all performance metrics (e.g., −0.994 with test accuracy), indicating that a reduction in model error corresponds to increased performance. Additionally, training accuracy and validation accuracy have high positive correlations with test accuracy (0.962 and 0.994, respectively), confirming the models' ability to generalize training data to new datasets effectively.

**TABLE 3:** Comparative performance evaluation of CNN models across configurations.

Models	Batch size	Image size	Test accuracy (%)	Test loss (%)	Precision (%)	Recall (%)	F1‐score (%)	Training accuracy (%)	Training loss (%)	Validation accuracy (%)	Validation loss (%)	Training time (sec)	GPU usage (MB)	Best epoch
VGG16	32	64 × 64	77	76.6	78.3	77	77	87.8	40.9	75.7	67.4	1388	78.885	82
		224 × 224	94.2	21.6	94.6	94.2	94.1	93.9	22.7	93	23.6	2092	172.957	89
	64	64 × 64	76.3	72.9	78.1	76.3	76.1	82.7	84.9	78.5	70.9	1281	77.304	99
		224 × 224	92.3	31.9	93	92.3	92.4	91.2	33	90.3	32	1889	140.862	98
EfficientNetB0	32	64 × 64	12.3	270.8	2.4	12.3	3.9	7.7	270.7	7	270.7	568	158.176	81
		224 × 224	7	270.8	0.9	7	1.6	6.7	270.8	6.7	270.8	970	45.458	7
	64	64 × 64	11.4	270.8	2.3	11.4	3.5	7	270.8	7.2	270.8	1526	24.349	70
		224 × 224	7	270.9	1	7	1.7	6	270.8	6.7	270.8	862	49.911	6
MobileNetV2	32	64 × 64	68.6	115.5	71.3	68.6	67.7	99.7	2.2	71	127.9	1084	16.241	74
		224 × 224	93	21.9	93.4	93	92.9	100	0.6	92.3	23.6	1096	17.095	91
	64	64 × 64	69.8	99.4	73.6	69.8	69.5	99	6.7	74	104.7	989	15.109	64
		224 × 224	95.2	13.5	95.5	95.2	95.1	99.9	1.6	95.9	11.9	624	34.754	74
DenseNet121	32	64 × 64	79.9	74.8	83.1	79.9	80.2	98	7.2	79.8	82.3	932	74.904	96
		224 × 224	97.3	8.3	97.6	97.3	97.3	99.7	2.1	97.3	7.1	1777	86.412	85
	64	64 × 64	81	61.3	82.9	81	79.7	96	15	77.6	83.1	893	72.493	86
		224 × 224	96.5	9.1	96.6	96.5	96.4	99	4.8	96.8	11.4	1597	114.488	99
ResNet50	32	64 × 64	32.1	207.6	30.3	32.1	28.4	35.2	193.9	35	200.7	2183	235.091	99
		224 × 224	39.5	178.5	50	39.5	35.7	38.8	177.1	39.4	179.8	1784	296.228	92
	64	64 × 64	27.2	215.8	28.7	27.2	24	31.6	206.1	25.1	215	1530	117.314	87
		224 × 224	41.4	174.9	49.2	41.4	37.9	38.7	182.8	42.2	171.1	1365	241.212	95
InceptionV3	32	75 × 75	73.1	115.5	75.3	73.1	72.7	99.6	2.6	74.4	111.4	2056	113.178	78
		299 × 299	95.1	13	95.3	95.1	95.1	99.8	1.2	93.5	24.5	2445	115.33	87
	64	75 × 75	69	125.8	72.1	69	68.6	98.7	6.8	72.5	118.2	1645	111.141	62
		299 × 299	91.7	26	92.3	91.7	91.7	99.6	2.7	90.5	28.7	1172	224.467	70

**TABLE 4:** Correlation among key performance metrics of evaluated CNN models.

	Test accuracy (%)	Test loss (%)	Precision (%)	Recall (%)	F1‐score (%)	Training accuracy (%)	Training loss (%)	Validation accuracy (%)	Validation loss (%)	Training time (sec)	GPU usage (MB)
Test loss (%)	−0.994**										
Precision (%)	0.993**	−0.985**									
Recall (%)	1.000**	−0.994**	0.993**								
F1‐score (%)	1.000**	−0.993**	0.994**	1.000**							
Training accuracy (%)	0.959**	−0.0929**	0.962**	0.959**	0.963**						
Training loss (%)	−0.955**	0.925**	−0.958**	−0.955**	−0.959**	−0.998**					
Validation accuracy (%)	0.998**	−0.989**	0.994**	0.998**	0.999**	0.968**	−0.96**				
Validation loss (%)	−0.992**	0.998**	−0.984**	−0.992**	−0.991**	−0.924**	0.919**	−0.989**			
Training time (sec)	0.228	−0.214	0.245	0.228	0.23	0.172	−0.17	0.233	−0.233		
GPU usage (MB)	−0.09	0.078	−0.035	−0.09	−0.092	−0.185	0.187	−0.092	0.061	0.440*	
Best epoch	0.513*	−0.504*	0.540**	0.513*	0.506*	0.436*	−0.416*	0.501*	−9.509*	0.384	0.377

*Significant at the 5% level. **Significant at the 1% level.

### 3.1 Model Architecture, Image Dimensions, and Batch Size

One of the most influential factors in classification performance is the choice of model architecture. For instance, the VGG16 model—with approximately 138 million parameters—achieved a test accuracy of 94.2% (test loss = 21.6%) when trained on images with a resolution of 224 × 224 and a batch size of 32. However, under the same batch configuration but with 64 × 64 inputs, its accuracy dropped significantly to 77.0% (test loss = 76.6%). This substantial performance difference aligns with findings from Nishio et al. (2018), who reported that larger image sizes yield better results, with validation accuracy increasing from 60.7% at 56 × 56 pixels to 68.0% at 224 × 224 using transfer learning. Similarly, Chansong and Supratid (2021) demonstrated that increasing image size from 50 × 50 to 150 × 150 improved accuracy by up to 2.71% in Conv573 architectures with varied kernels. These studies reinforce the explanation that higher‐resolution inputs provide richer visual information, enabling the network to better detect fine details such as seed shapes, textures, and other subtle patterns critical for classification tasks. Specifically, in successful models like VGG16 and DenseNet, the increased resolution facilitated stronger feature extraction and allowed the networks to make finer distinctions between subtle class characteristics, thereby directly contributing to higher generalization capabilities.

In contrast, DenseNet121—with only 8 million parameters—achieved an impressive 97.3% accuracy (test loss = 8.3%) using 224 × 224 images and a batch size of 32. This performance is consistent with findings from Yurdakul et al. (2024), where DenseNet121 demonstrated superior results in almond variety classification with 99.16% accuracy, 99.17% precision, and 99.16% recall, highlighting its repeated feature reuse across layers. This architecture demonstrated a superior balance between model complexity and performance, effectively mitigating overfitting and ensuring robust generalization on the test set. Moreover, EfficientNetB0, despite its optimized compound scaling and only about 5.3 million parameters, performed very poorly: its accuracy was only 12.3% (test loss ≈ 270.8%) at 64 × 64 resolution and 7.0% (with the same loss) at 224 × 224. This unexpected failure suggests a critical issue with generalization rather than model capacity. While EfficientNet is typically a top‐tier performer, its poor results here indicate a severe susceptibility to domain shift—the disparity between the natural scenes in pre‐training data and our specific macro seed imagery with black backgrounds. Furthermore, unlike MobileNet or DenseNet, EfficientNet proved highly sensitive to the uniform hyperparameters used in this study. It likely suffered from optimization difficulties, such as vanishing gradients or an inappropriate learning rate for its specific depth‐wise separable convolutions, which prevented the model from converging. This confirms that without architecture‐specific tuning, its theoretical capacity does not guarantee generalization in practice.

Meanwhile, MobileNetV2—with 3.4 million parameters and inverted residual blocks—struck an effective balance between accuracy and efficiency. At 224 × 224 resolution and batch size 32, it achieved 93.0% accuracy (test loss = 21.9%), which improved to 95.2% (test loss = 13.5%) when the batch size was increased to 64, while significantly reducing training time and consuming only 17 to 35 MB of GPU memory. This improvement with larger batch size partially contradicts findings from Kandel and Castelli (2020), who observed that batch size 32 yielded slightly higher accuracy (91.1%) compared to batch size 64 (91.0%) at epoch 75, suggesting that the impact of batch size can be architecture‐ and task‐dependent. However, MobileNetV2's efficiency is further validated by Xu et al. (2022), who reported a 98.58% accuracy and 97.57% F1‐score for MobileNet in rapid maize seed classification, positioning it as a robust choice for resource‐constrained settings. In comparison, ResNet50—with 25.6 million parameters and residual blocks—showed average results (accuracy of 32.1% at 64 × 64 with batch size 32, increasing to 41.4% at 224 × 224 with batch size 64) and incurred high computational costs (up to 2183 s of training time and 296 MB of GPU memory usage), reflecting its complexity and limited suitability for real‐time applications. This suboptimal performance, despite the model's depth, further emphasizes optimization challenges. The distinct lack of convergence suggests that ResNet, similar to EfficientNet, required more aggressive regularization or specific data scaling to overcome the vanishing gradient problem in this specific domain, leading to limited generalization compared to the highly efficient DenseNet121.

Furthermore, the InceptionV3 model—featuring multi‐branch modules and 23.9 million parameters—achieved 95.1% accuracy (test loss = 0.130) at 299 × 299 resolution with batch size 32, but its accuracy dropped to 73.1% (test loss = 1.155) at 75 × 75. This trend corroborates the findings of Laabassi et al. (2021), who reported a high test accuracy of 95.62% for InceptionV3 in wheat variety classification, emphasizing the model's dependence on higher‐resolution inputs for optimal performance. Therefore, when high‐quality images are available, using 299 × 299 resolution is recommended for InceptionV3 to achieve superior classification results. These results collectively demonstrate that increasing input image resolution consistently improves model performance and reduces test loss across architectures tested in this study. For instance, using higher‐resolution images (224 × 224 for most models and 299 × 299 for InceptionV3) significantly increased accuracy: from about 77% to 94.2% in VGG16, from 79.9% to 97.3% in DenseNet121, and from 73.1% to 95.1% in InceptionV3. This improvement is reasonable from a computer vision standpoint; higher resolution allows for more accurate extraction of visual patterns and reduces intermediate feature loss. Consequently, models like VGG16, DenseNet121, and InceptionV3 could leverage these details to draw finer distinctions between classes, enhancing their ability to generalize to unseen data—a perspective supported by Pratiwi et al. (2021) with their reported accuracy gains of up to 5.4% with increased input size. Additionally, the notable decrease in test loss and the increase in precision and recall (e.g., a drop from 74.8 to 8.3 and a rise to 97% in DenseNet121) indicate stronger feature extraction capabilities and a significantly reduced risk of overfitting in these high‐performing architectures.

Batch size optimization is also of great importance—especially considering hardware limitations and the model's sensitivity to gradient updates. Different chosen batch sizes may lead to varying accuracies in testing and training, as well as different runtimes. In our study, a batch size of 32 generally yielded better results compared to a batch size of 64, particularly for heavier models where smaller batches can act as a regularizer and help the model escape sharp minima, thereby improving generalization. Lin (2022) suggests that mini‐batch sizes between 16 and 64 are optimal for achieving high accuracy and efficiency in CNN training, with a starting point of 32 often being ideal, which aligns with our results while also corresponding to the improved performance of MobileNetV2 at a batch size of 64 (95.2% accuracy). However, Magboo and Abu (2023) found that smaller batch sizes generally outperformed larger ones, indicating that the optimal batch size may vary depending on the specific application and dataset. Moreover, training time and GPU memory usage are critical factors when selecting models for industrial or real‐time applications. For example, while DenseNet121 ranks among the top models in terms of test accuracy (up to 97.3%), its training time at 64 × 64 resolution (e.g., 893 or 932 s) differs significantly compared to its higher‐resolution version. In contrast, MobileNetV2 recorded training times of 1084 s (batch size 32) and 624 s (batch size 64) at 224 × 224 resolution, making it a highly suitable option for real‐time or resource‐constrained environments, effectively balancing computational cost with high generalization performance. As further evidenced by its strong performance metrics (95.11% accuracy, 96.46% precision) in almond variety classification (Yurdakul et al. 2024), MobileNetV2 demonstrates that efficient architecture design can achieve competitive results without the massive parameter count that often hinders the training stability of larger models like ResNet50 in limited‐data scenarios.

In summary, the results clearly show that increasing the input image resolution consistently improves model performance. This higher accuracy is pivotal for detecting even minute morphological differences that signal adulteration, thereby directly enhancing food‐safety monitoring and consumer protection. Additionally, batch‐size optimization remains a critical aspect of model training, as its influence on performance can vary depending on the architecture and data characteristics. When properly tuned, an optimal batch size not only boosts accuracy but also shortens inference time, enabling rapid on‐site screening at customs and industry checkpoints—an essential requirement for maintaining trade integrity and preventing the circulation of mislabeled or fraudulent products. Therefore, selecting the appropriate model architecture, image input size, and batch size is essential for achieving optimal classification performance and for deploying a practical, field‐ready system that safeguards the authenticity of food‐related medicinal Apiaceae species.

### 3.2 Confusion Matrix Analysis

**FIGURE 3:**
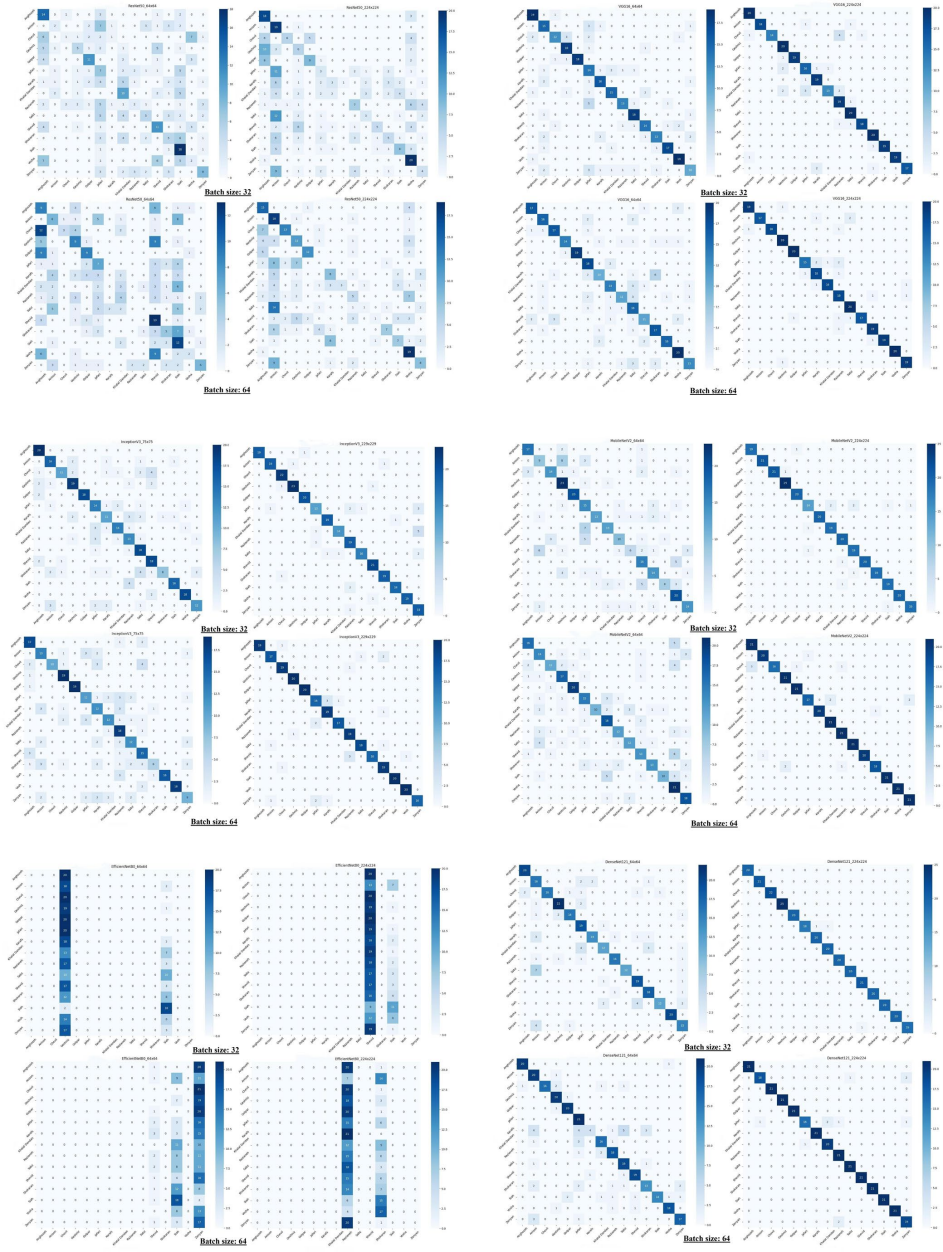
Confusion matrices of CNN models across different batch sizes and input image resolutions, illustrating classification accuracy and inter‐class prediction errors.

To evaluate the classification performance and identify specific misclassification patterns among Apiaceae species, confusion matrices were generated for all experimental scenarios (Figure 3). While overall accuracy metrics provide a general performance summary, the confusion matrix offers a granular view of the model's ability to distinguish between morphologically similar species. A systematic analysis of the matrices reveals that increasing image resolution was the most critical factor in reducing inter‐class confusion. For instance, at the lower resolution of 64 × 64 pixels, the MobileNetV2 model exhibited significant confusion between Shevid ( *Anethum graveolens* ) and seven other visually similar species. However, upscaling the input to 224 × 224 pixels completely eliminated these false positives, enabling the model to distinguish these species without error, regardless of batch size variations (32 or 64). A similar trend was observed with the DenseNet121 model; low‐resolution inputs led to substantial confusion between Zireh Siah ( *Carum carvi* ) and several other taxa. Conversely, high‐resolution inputs allowed the model to accurately differentiate these species. Other high‐performing models demonstrated distinct strengths: VGG16 minimized confusion for Shokaran ( *Conium maculatum* ) and Vosha (*Ferula ammoniacum*), while InceptionV3 showed minimal confusion for Golpar (*Heracleum persicum*) at higher resolutions.

Despite the general improvement with resolution, architectural differences played a significant role. While MobileNetV2 and DenseNet121 achieved near‐perfect separation, the ResNet50 model struggled to fully distinguish between species, showing only marginal improvements at higher resolutions. Furthermore, the EfficientNetB0 model proved unsuitable for this specific dataset, failing to differentiate species effectively across all tested parameters. These visual observations from the confusion matrices corroborate the quantitative metrics presented in Table 3. For example, MobileNetV2 (224 × 224, batch size 64) achieved a test accuracy of 95.2% and an F1‐score of 95.1%, while DenseNet121 (224 × 224, batch size 32) reached a test accuracy of 97.3%. In stark contrast, EfficientNetB0 recorded a negligible test accuracy of 7%, confirming the high off‐diagonal values observed in its confusion matrix.

The misclassifications observed, particularly at lower resolutions, are biologically grounded. All studied seeds belong to the Apiaceae family and share a characteristic schizocarpic structure (Lucena et al. 2001), resulting in high morphological and micromorphological similarity (Plunkett et al. 2018). This resemblance is the primary driver of misidentification and fraud in commercial markets (Colombo et al. 2009; Welke et al. 2015). The superior performance of high‐resolution models suggests that distinguishing these cryptic species requires the extraction of fine‐grained features—such as subtle texture variations, striation patterns, and surface protrusions—which are lost in down‐sampled images (Pratiwi et al. 2021).

Overall, confusion matrices are essential tools for evaluating classification models. They enable researchers to pinpoint weaknesses in a model's ability to distinguish between visually similar species. Even the most precise models may fail when confronted with highly similar traits. Crucially, each misclassification cell maps to a concrete food‐safety or trade‐integrity risk: mislabeling a toxic species as edible jeopardizes consumer health, whereas replacing a high‐value spice with a cheaper look‐alike constitutes economic fraud. Insights from these matrices inform better feature‐extraction strategies, such as architectural adjustments, input‐resolution selection, and batch‐size tuning. By systematically reducing the error rates highlighted in the confusion matrices, the proposed CNN framework promotes regulatory compliance, prevents adulteration, and safeguards supply‐chain transparency. For instance, if major errors occur between commercially sensitive species like green cumin and toxic plants such as poison hemlock, data augmentation or transfer‐learning‐based hybrid models can be employed to capture subtle differences. These targeted refinements not only boost model accuracy but also yield practical, field‐ready diagnostics that protect consumer health and uphold trade authenticity.

### 3.3 Learning Dynamics

**FIGURE 4:**
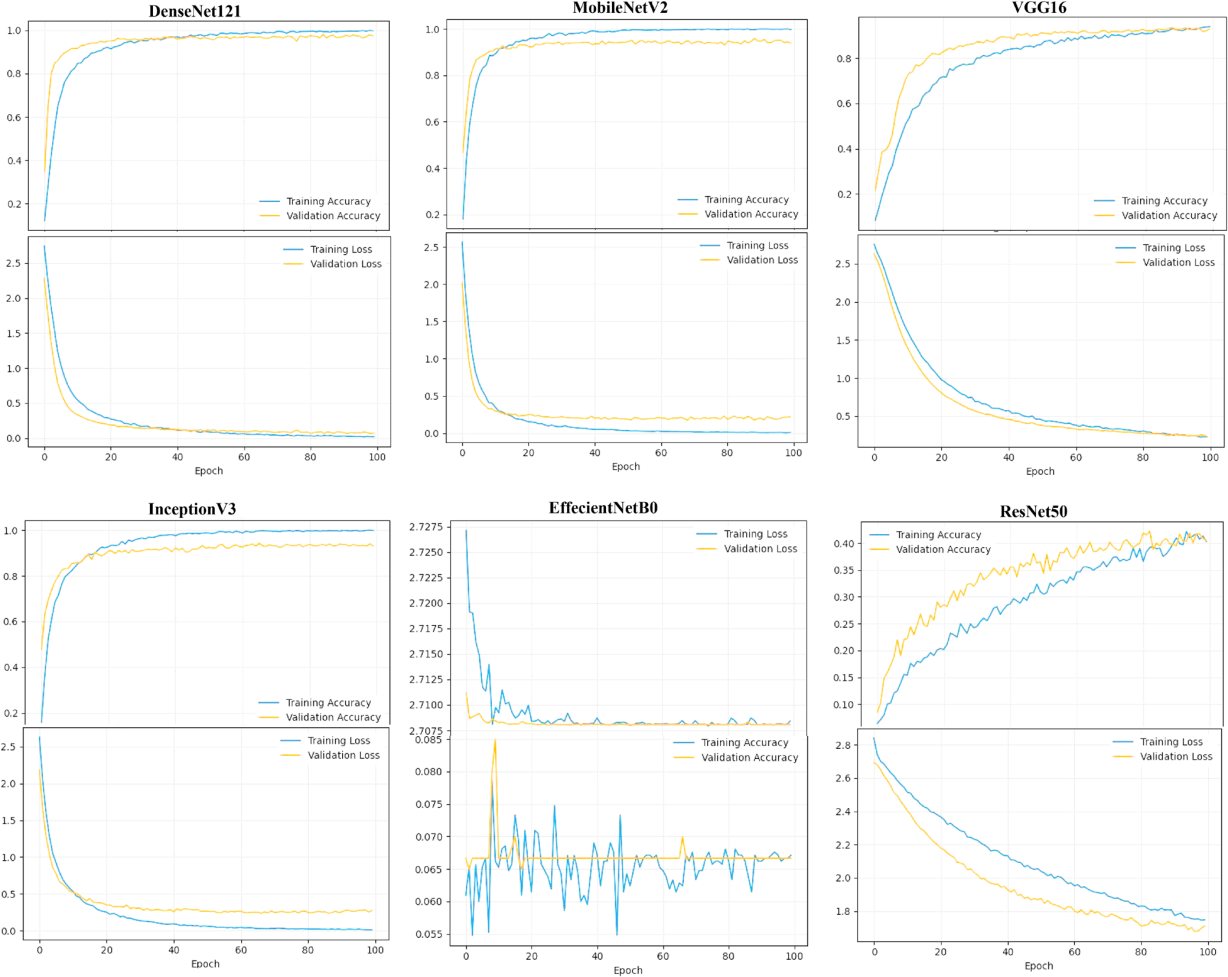
Learning dynamics of CNN models: Training and validation accuracy/loss curves across epochs, highlighting convergence behavior and overfitting patterns.

Analyzing the learning dynamics of models during training is critical for accurately assessing the training process in deep learning models—particularly convolutional neural networks (CNNs), which are widely used in image processing (Figure 4). Selecting an optimal CNN architecture for a specific computer vision task requires more than merely evaluating final performance metrics such as test accuracy (Yurdakul et al. 2024). Accuracy and loss curves plotted across training epochs offer valuable insight into the model's learning dynamics, behavior on training and validation data, stability, convergence rate, generalization ability, and susceptibility to undesirable phenomena such as overfitting or underfitting (Attri et al. 2023; El Sakka et al. 2025). Given that higher resolution and a batch size of 32 consistently led to optimal performance, stability, and computational efficiency in most models (such as VGG16, MobileNetV2, DenseNet121), the analysis of accuracy and loss graphs was conducted under these optimal conditions. The goal was to provide a comprehensive assessment of how these models learn, highlighting their intrinsic strengths and weaknesses in this specific application context—thereby offering guidance for model selection in future research.

#### 3.3.1 The High Performing Models

DenseNet121, MobileNetV2, InceptionV3, and VGG16—exhibited effective and stable learning behaviors that resulted in strong validation and test performances. However, detailed examination of their learning curves revealed notable distinctions in their optimization dynamics (Figure 4). DenseNet121 demonstrated outstanding performance characterized by rapid and smooth convergence. Within the first 25 epochs, validation accuracy surged and loss dropped sharply. A well‐defined plateau followed between epochs 60–80, with final validation accuracy reaching ~97% and validation loss falling to ~7%. Notably, the gap between training and validation curves remained consistently small, indicating very mild overfitting and high generalization ability. This behavior is consistent with the architectural principle of dense connectivity in DenseNet, where each layer receives the feature maps of all preceding layers (Huang et al. 2017). By encouraging extensive feature reuse and providing short paths for gradient flow, DenseNet121 can quickly learn a rich set of low‐ and mid‐level features relevant to seed texture, shape, and color, while maintaining stable optimization. The dense connections also reduce the risk of vanishing gradients, which helps explain the smooth convergence and the limited discrepancy between training and validation performance. In our implementation, these architectural advantages are further supported by the transfer‐learning setup (frozen ImageNet feature extractor plus a regularized classification head), enabling DenseNet121 to adapt its generic visual features efficiently to the fine‐grained seed traits (Huang et al. 2017; Yurdakul et al. 2024). The test accuracy of 97.3% further confirmed the model's robustness.

MobileNetV2 also achieved strong results with a very fast early learning phase (10–20 epochs). Training accuracy quickly neared 100%, and validation accuracy reached ~92%. Although the training–validation gap was slightly larger than in DenseNet121, it remained controlled. These dynamics reflect the lightweight design of MobileNetV2, which relies on inverted residual blocks with linear bottlenecks and depthwise separable convolutions (Sandler et al. 2018). Depthwise separable convolutions drastically reduce the number of parameters and computations, allowing the network to learn discriminative filters for seed patterns very efficiently, which manifests as the rapid rise in training accuracy. At the same time, the constrained capacity and compressed feature representations in the bottleneck layers can make the model somewhat more sensitive to subtle intra‐class variability and noise, leading to a slightly larger gap between training and validation curves and minor fluctuations in the later epochs. In our transfer‐learning framework with a shared augmentation pipeline, this trade‐off between efficiency and representational capacity explains why MobileNetV2 reaches strong but slightly less stable performance compared to DenseNet121.

Exhibiting even more robust convergence patterns, InceptionV3 displayed highly efficient learning dynamics, achieving peak performance by epoch 87. Training accuracy reached 99.8%, and validation accuracy stabilized around 93.5%, with minimal losses (~1.3% training, ~24.5% validation). The model showed rapid convergence and maintained stability throughout, consistent with its multi‐scale feature extraction design that processes spatial hierarchies efficiently (Szegedy et al. 2016). Specifically, the Inception modules apply multiple receptive field sizes (e.g., 1 × 1, 3 × 3, 5 × 5) in parallel, allowing the network to learn fine‐scale texture patterns on the seed coat (such as speckles and subtle surface irregularities) together with coarser features like overall seed outline, symmetry, and boundary shape within the same layer (Richter et al. 2022). This multi‐branch, multi‐scale processing is particularly advantageous for our dataset, where several species differ only by subtle combinations of textural and shape cues across the adaxial and abaxial seed surfaces. Moreover, the factorized convolutions and extensive use of batch normalization in InceptionV3 stabilize gradient flow and reduce internal covariate shift, which helps explain the fast yet smooth convergence and the relatively stable validation accuracy observed over epochs (Ioffe and Szegedy 2015). Coupled with our transfer‐learning strategy and regularized top layers, these architectural properties directly support the model's ability to adapt pre‐trained multi‐scale filters to the fine‐grained seed morphology. Even with a different batch size (64), the model maintained performance (91.7% test accuracy), indicating robustness. Overall, these architectural properties link the outstanding performance of InceptionV3 directly to its feature learning dynamics, whereby multi‐scale and factorized convolutions enable effective modeling of fine‐grained inter‐species differences in seed morphology.

Finally, VGG16 exhibited a successful, albeit slower, learning process. In the first 30 epochs, validation accuracy surpassed training accuracy—a likely consequence of strong regularization techniques like dropout embedded in its classical deep architecture (Simonyan and Zisserman 2014). The model gradually converged between epochs 80–90, ending with training and validation accuracies of 93.9% and 93%, respectively. The narrow training–validation gap suggested minimal overfitting. This pattern can be attributed to the simple yet deep stack of 3 × 3 convolutional layers and max‐pooling operations in VGG16, which encourages the progressive extraction of hierarchical features from local edge and texture cues to more global shape descriptors (Simonyan and Zisserman 2014). When combined with dropout and other regularization strategies in the fully connected layers, the model may initially underfit the training data, causing the validation accuracy to appear slightly higher in early epochs. As training proceeds, VGG16 slowly fits both training and validation distributions while retaining good generalization, which is reflected in its stable late‐epoch behavior and limited overfitting. Within our unified transfer‐learning and augmentation framework, this explains why VGG16, despite its older and parameter‐heavy design, ultimately reaches competitive test accuracy while maintaining a controlled generalization gap. The test accuracy of 94.2% confirms that VGG16, despite being an older design, retains excellent generalization when trained properly. These observations support previous findings that architectural principles—dense connections, residuals, multi‐scale processing, and regularization—play critical roles in training stability and generalization (Huang et al. 2017; Sandler et al. 2018; Szegedy et al. 2016; Simonyan and Zisserman 2014).

#### 3.3.2 Underperforming Models

EfficientNetB0 and ResNet50‐ in contrast to the four successful models, performed poorly in these experiments, with their learning curves clearly reflecting this failure. EfficientNetB0 presented a classic case of complete learning failure and severe underfitting. Both training and validation accuracies remained extremely low (~6%–8%) across all 100 epochs—comparable to random guessing. Losses were exceedingly high (~270%) and showed no meaningful decline. The training accuracy curve was highly erratic and unstable, indicating severe instability in the optimization process. No significant gap between training and validation curves was observed, which, rather than indicating overfitting, highlighted the model's inability to learn any meaningful patterns from even the training data. These poor outcomes were consistent across all tested configurations (as reflected in the performance table), suggesting an issue deeper than a specific hyperparameter setting. Likely incompatibility between EfficientNetB0's compound scaling strategy and the dataset's structure, as EfficientNet models are highly sensitive to scale‐related hyperparameters (Tan and Le 2019).

Similarly, ResNet50 exhibited underwhelming performance, albeit not as poor as EfficientNetB0. The learning curves indicated significant underfitting and an incomplete learning process. Final training accuracy only reached ~41.8%, with validation accuracy at 40.5% and test accuracy at 36.8%. Losses remained high (~175% for training and 168% for validation). A particularly unusual pattern was observed: validation accuracy consistently exceeded training accuracy, and validation loss remained lower than training loss through much of the training. As discussed in the individual analysis, this may be attributable to strong regularization effects or statistical discrepancies between training and validation sets. Nonetheless, the overall performance was far below expectations for such a well‐established architecture. The absence of a convergence plateau, even after 100 epochs, suggests that the model either required significantly more training or that its hyperparameters (such as learning rate) were suboptimal tuning (Goodfellow et al. 2016; Bishop and Nasrabadi 2006; Bergstra and Bengio 2012). The relatively high fluctuation in its learning curves further reflected a lack of training stability compared to the successful models. In contrast, ResNet50 exhibited partial underfitting, achieving only ~41.8% training accuracy and ~36.8% test accuracy. Interestingly, validation accuracy remained slightly higher than training accuracy for much of the training, a pattern that can be attributed to over‐regularization or batch norm behavior in shallow training regimes (He et al. 2016). Furthermore, the model's loss curves failed to converge even after 100 epochs, and learning remained unstable—likely due to suboptimal learning rate settings, a common issue in training deep residual networks (Smith 2017). These observations indicate that while both models have strong architectural foundations, their training requires careful tuning and may not be inherently suited to the dataset used in this study without adaptation.

This comparative analysis of the learning dynamics across six CNN architectures reveals several key insights. The outstanding performance of the four successful models—DenseNet121, MobileNetV2, InceptionV3, and VGG16—confirms that deep CNNs can effectively learn from this dataset, although architectural choice significantly impacts success. DenseNet121 emerged as the best‐performing model, combining excellent final performance, stable convergence, and minimal overfitting. MobileNetV2 and InceptionV3 also performed strongly, with InceptionV3 showing the fastest convergence and both models maintaining controlled overfitting. VGG16, despite being an older architecture, achieved good performance but converged more slowly and exhibited a greater tendency toward overfitting. Importantly, all these models were trained under a unified experimental protocol—identical preprocessing, data augmentation, optimizer settings, and a common transfer‐learning head—ensuring that the observed differences in learning dynamics primarily reflect the intrinsic architectural characteristics summarized in Table 2, rather than inconsistencies in implementation.

In contrast, EfficientNetB0 and ResNet50 underperformed, demonstrating that architectural sophistication alone does not ensure success; instead, proper hyperparameter tuning, preprocessing compatibility, and pretrained weight quality are critical. Severe underfitting, especially in EfficientNetB0, highlights the need for deeper investigation into model‐data compatibility and training configuration. The analysis of accuracy and loss curves underscores the importance of examining generalization gaps—while all successful models showed some overfitting, the key was maintaining a stable and non‐divergent gap, which may serve as an indicator of a model's inherent tendency toward overfitting (VGG16 > MobileNetV2/InceptionV3 > DenseNet121). Convergence speed also varied notably, with InceptionV3 converging fastest (~epoch 66–70), followed by DenseNet121 and MobileNetV2 (~epoch 88), and VGG16 being the slowest (~epoch 94), which is critical in scenarios with limited computational resources or training time. Overall, this comprehensive analysis underscores that model efficiency, convergence behavior, and generalization capability are jointly determined by the interplay between the backbone architecture (Table 2) and our implementation strategies—namely standardized transfer learning, a shared regularized classification head, and consistent augmentation. Beyond mere technical evaluation, these findings carry significant practical implications: superior models like DenseNet121 and InceptionV3 offer more reliable adulteration detection, directly protecting consumer health from toxic look‐alikes (e.g., poison hemlock) while preserving the economic value of premium spices (e.g., green cumin). By minimizing misclassification rates, the proposed framework supports regulatory compliance, enhances food‐safety surveillance, and safeguards trade integrity across the medicinal‐plant supply chain. Ultimately, this integrated perspective extends beyond quantitative metrics, offering actionable guidance for informed model selection in both applied and research contexts.

### 3.4 t‐SNE Graph Analysis

**FIGURE 5:**
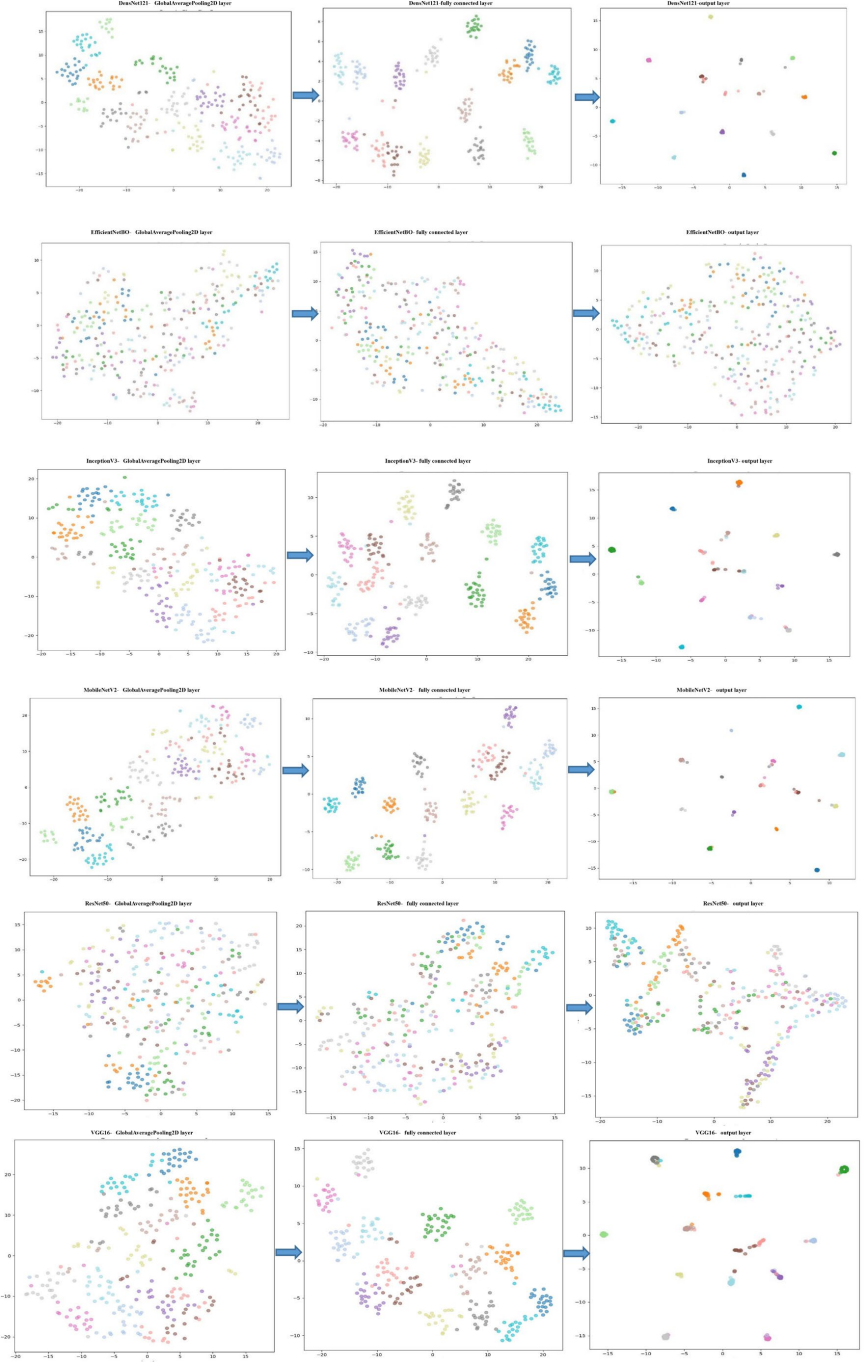
t‐SNE visualization of feature representations across CNN layers, illustrating species‐specific clustering and separability in the latent feature space. Color code: Anghozeh (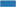), Anison (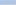), Chevi (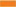), Geshniz (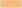), Golpar (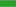), Jafari (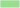), Karafs (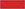), Khalal Dandan (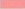), Razianeh (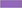), Zireh Sabz (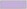), Shevid (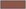), Shokaran (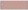), Zireh Siah (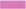), Vosha (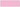), Zenyan (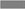).

This analysis, which explores how feature representations evolve as they propagate through the layers of deep convolutional neural networks (CNNs), offers critical insight into each model's capacity to extract and isolate diagnostic features across multiple species. By tracing this progression from raw input to deep representations, we can better understand how models construct high‐level semantic abstractions and decision boundaries. At the input stage, raw hyperspectral or multispectral data are mapped into a high‐dimensional feature space. However, t‐SNE visualizations of these representations show substantial overlap between species classes, with no clear boundaries (Figure 5). This reflects the insufficient discriminative power of raw spectral signals for species‐level classification, as has been highlighted in recent deep learning applications to biological and spectral data (Ching et al. 2018). It underscores the necessity of deep hierarchical processing to extract class‐specific features from noisy and entangled inputs. As inputs pass through the convolutional layers, the networks begin identifying informative features. Early layers tend to extract low‐level elements (e.g., edges, textures), yielding slight separation in t‐SNE plots. Deeper layers further refine these features, enabling more distinct species clusters.

Among the evaluated models, DenseNet121 demonstrates the most effective feature progression. Its densely connected architecture promotes efficient gradient flow and feature reuse, resulting in early and sharp cluster separations even in mid‐layer t‐SNE plots (Huang et al. 2017). By the output layer, it achieves complete class segregation for all 15 species, highlighting its superior discriminative capability. Similarly, MobileNetV2, with its lightweight and inverted residual design, shows gradual separation of species clusters. Although its final clusters are less distinct than DenseNet121's, it still yields clear, consistent groupings, reflecting effective feature compression and transformation (Sandler et al. 2018). InceptionV3, known for multi‐branch and multi‐scale feature extraction, achieves notable separation already in mid‐layers. Its architectural diversity allows simultaneous processing of multiple receptive fields, contributing to rapid development of distinct class clusters (Szegedy et al. 2016). Final‐layer t‐SNE plots confirm clean, well‐defined clusters for nearly all species. VGG16, in contrast, reflects slower feature development due to its traditional sequential structure. Although meaningful separation appears in mid‐layers, its final output clusters remain partially overlapping, consistent with it's relatively lower classification performance and higher overfitting risk (Simonyan and Zisserman 2014).

Conversely, EfficientNetB0 fails to produce well‐separated clusters across all layers. Despite its compact architecture and compound scaling, it lacks sufficient feature resolution to differentiate species effectively—likely due to sensitivity to scale‐related hyperparameters and limited representational capacity (Tan and Le 2019). ResNet50, although designed with deep residual connections, also exhibits poor t‐SNE separation. Minor clustering may appear mid‐way, but final representations remain highly entangled, indicating convergence or optimization inefficiencies during training (Smith 2017). Collectively, these t‐SNE analyses underscore that effective species classification depends not merely on final metrics but on a model's ability to extract and refine discriminative deep features through hierarchical convolutional processing. Models like DenseNet121, MobileNetV2, and InceptionV3 succeed in generating clear and separable semantic clusters, while others—EfficientNetB0 and ResNet50—struggle to achieve similar representational clarity. Future research can benefit from these insights by focusing on optimizing convolutional paths, adopting advanced fine‐tuning, and leveraging ensemble and transfer learning strategies to reduce cluster overlap. Furthermore, detailed t‐SNE analyses can serve as a diagnostic tool to guide model selection and training improvements in spectral and botanical classification applications. Ultimately, the in‐depth examination of t‐SNE graphs and multi‐model comparisons paves the way for novel research avenues toward developing more efficient and precise plant classification systems—offering significant value both scientifically and practically.

### 3.5 Visualization of Hierarchical Feature Maps

**FIGURE 6:**
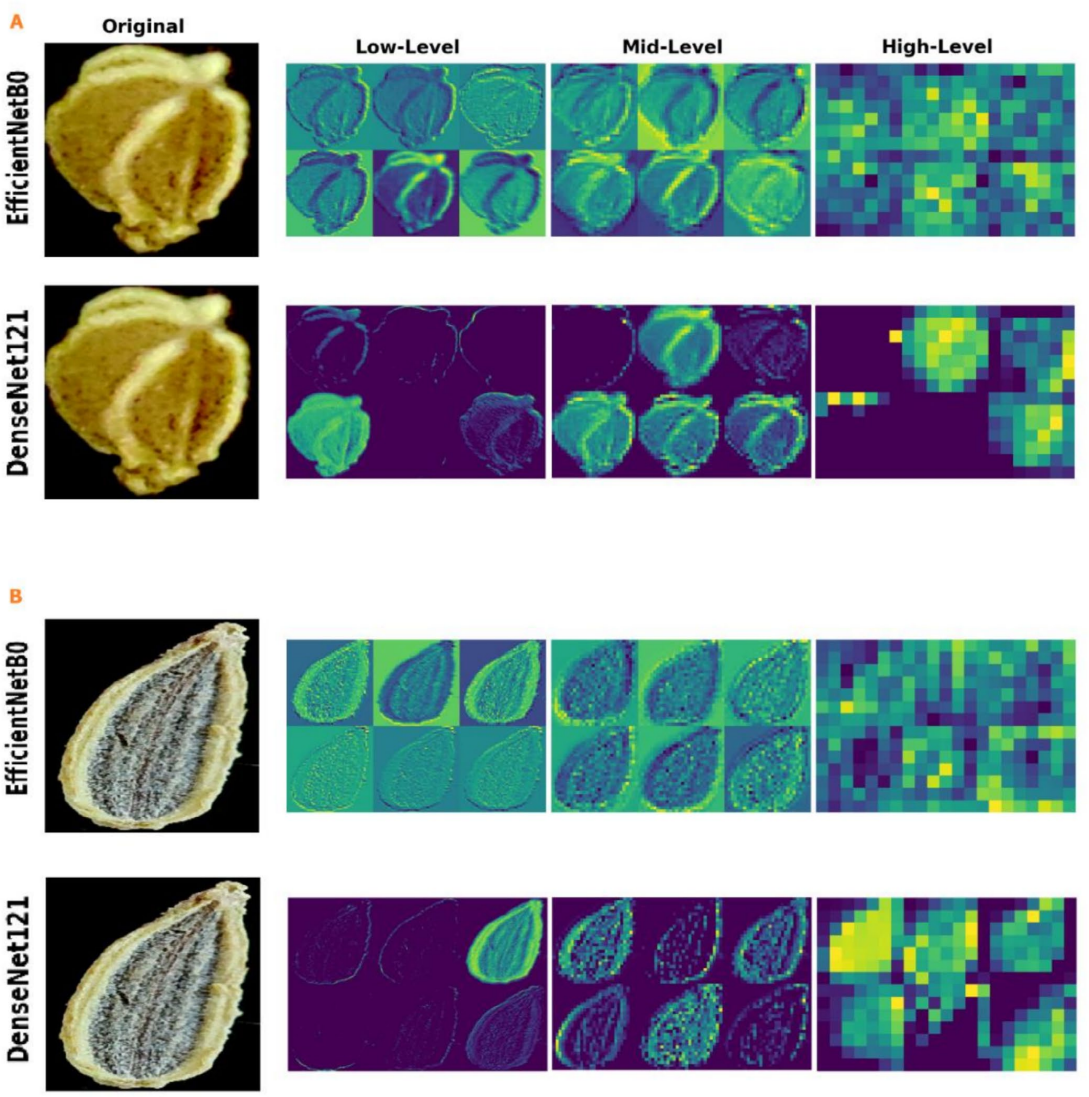
Hierarchical feature visualization of seed images: (A) celery ( *Apium graveolens* ) seed and (B) fennel ( *Foeniculum vulgare* ) seed.

To better understand the learning mechanisms of the tested models—specifically, the underperforming EfficientNetB0 versus the outperforming DenseNet121—and to ensure that predictions rely on biologically meaningful traits rather than image artifacts, we analyzed their internal representations using feature maps (Figure 6). This hierarchical analysis showed that representation quality is the primary determinant of feature separability and overall performance in fine‐grained Apiaceae seed classification. In both networks, low‐level feature maps from early convolutional layers function as edge and texture detectors: they highlight peripheral contours, surface roughness, and fine rugosity while suppressing homogeneous regions (Zeiler and Fergus 2014). However, the trajectory of feature evolution—from primitive cues (e.g., radial furrows, morphological symmetries, and simple texture patterns) to high‐level semantic concepts—is more coherent and better preserved in DenseNet121. Qualitative comparison indicates that DenseNet121 retains subtle geometric and textural characteristics with greater clarity even in the early hierarchy, which is essential for discriminating phenotypically similar species. By contrast, EfficientNetB0 produces faint and diffuse maps at mid‐level stages, reflecting a failure to aggregate initial cues into discriminative intermediate representations—an weakness that directly explains its poorer fine‐grained performance.

At mid and high abstraction levels, the disparity becomes more pronounced. DenseNet121 exhibits strong sensitivity to high‐frequency details and selectively concentrates on diagnostic seed structures in a tight, biologically meaningful manner—particularly around the hilum, seed coat ornamentation, longitudinal ridges (costae), the overall seed curvature, and peripheral boundaries. Such localized, semantically aligned activation suggests that the model is not exploiting trivial cues such as background color, illumination artifacts, or image borders, but instead relies on species‐specific morphological markers (Selvaraju et al. 2017). This spatial focus therefore validates the decision logic of the top‐performing model. In sharp contrast, EfficientNetB0 shows weak, non‐localized, and diffuse patterns that often spread into irrelevant background areas, implying reliance on spurious correlations or non‐discriminative cues and matching the observed low quality of its mid‐level feature maps. Moreover, for species pairs with higher confusion, even the more accurate model may concentrate on partially overlapping morphological regions, indicating that misclassifications primarily stem from genuine phenotypic similarity rather than irrelevant features.

The origin of this performance gap is rooted in architectural differences. DenseNet121, through dense connections and feature reuse, enables edge and texture information to propagate from shallow to deep layers with minimal degradation—crucial for biological imagery where classification signals reside in anatomical structures and surface micro‐patterns (Litjens et al. 2017; Huang et al. 2017). In contrast, EfficientNetB0's MBConv blocks and compound scaling, while advantageous for information compression and computational efficiency, can induce premature loss of precise spatial detail, which is evident in the overly smooth and abstract feature maps even at relatively early stages (Tan and Le 2019; Sandler et al. 2018). These qualitative observations align with quantitative trends: EfficientNetB0's underperformance corresponds to its inability to build a well‐separated feature space, visually echoed by its diffuse feature maps, whereas DenseNet121's superiority is attributable to its clearer, semantically grounded representations that better preserve discriminative seed structures. Overall, the clarity, quality, and semantic alignment of extracted mid‐ and high‐level features emerge as the primary determinants of final accuracy, supporting the conclusion that dense‐connection architectures offer a substantial advantage for fine‐grained classification of seeds with complex surface structures compared with models optimized mainly for computational efficiency.

## 4 Conclusion

This study presents a multidimensional and in‐depth evaluation of convolutional neural networks for classifying mericarp seeds of medicinal Apiaceae species—an intricate task involving taxonomic ambiguity, morphological convergence, and a high risk of economically motivated adulteration that can jeopardize consumer health. By integrating not only final classification metrics but also learning dynamics, confusion matrices, and t‐SNE feature space visualizations, this research provides a comprehensive, diagnostically rich framework for understanding how CNNs extract, differentiate, and abstract semantic features through hierarchical depth. Among the tested models, DenseNet121 emerged as the most robust, achieving outstanding accuracy, stable convergence, and visually distinct species separation due to its densely connected architecture. MobileNetV2 and InceptionV3 also demonstrated high efficiency and discriminative power, particularly in resource‐constrained environments. However, models like VGG16, while accurate, suffered from slower convergence and higher risk of overfitting. EfficientNetB0 and ResNet50 highlighted a critical insight: architectural complexity alone does not ensure performance unless aligned with data structure, preprocessing, and hyperparameter tuning. Our empirical diagnostics—including erratic learning curves (Figure 4), severely high loss, entangled t‐SNE representations (Figure 5), and diffuse feature maps (Figure 6)—demonstrate that these architectures, under our standardized protocol, experienced profound optimization instability and representational failure, consistent with domain‐shift sensitivity. The study further emphasizes that image resolution had a greater impact than batch size on model accuracy. Specifically, upgrading from 64 × 64 to 224 × 224 resolution consistently improved test accuracy and reduced loss across top‐performing models (e.g., DenseNet121 from 0.799 to 0.973). While higher resolution increased memory usage, optimal batch sizes (e.g., 32 or 64) offered trade‐offs between computational efficiency and performance; these resource considerations are vital for real‐time quality‐control stations at customs or processing plants. In some cases, even training time decreased, as observed in InceptionV3. Complementing these results, hierarchical feature‐map inspection confirmed that DenseNet121's superior accuracy stems from clearer, tightly localized, and morphologically aligned internal representations, whereas weaker models exhibited diffuse mid‐level activations indicative of reduced discriminative capacity. We acknowledge that architecture‐specific hyperparameter tuning and low‐level gradient diagnostics (e.g., learning rate sweeps) could offer deeper mechanistic insights; we have incorporated this perspective as a direction for future work. Crucially, the reduced misclassification rates achieved by the best models translate into tangible public‐health and economic benefits: accurate discrimination prevents unintended inclusion of toxic look‐alikes (e.g., poison hemlock), preserves the market value of premium spices (e.g., green cumin), and strengthens regulatory compliance across the medicinal‐plant supply chain. These findings advocate for a paradigm shift from metric‐centric evaluation to interpretive, visually guided model selection—where semantic separability, convergence behavior, and computational balance coalesce to support food‐safety surveillance, consumer protection, and trade integrity. This study also underscores the pragmatic value of controlled comparative evaluation under uniform training conditions—a methodological approach that provides decisive, real‐world insights for applied settings where extensive per‐model tuning is often impractical. Future research should explore adaptive pipelines incorporating transfer learning, ensemble modeling, and targeted data augmentation to enhance generalization. Ultimately, this research affirms CNNs as transformative tools in botanical informatics, providing a replicable, field‐ready blueprint for real‐world authentication systems that couple high accuracy with transparent interpretability.

## Author Contributions

**Elyas Aryakia:** conceptualization, investigation, methodology, software, data curation, supervision, formal analysis, project administration, writing – review and editing, writing – original draft, funding acquisition, validation, visualization, resources. **Ersam Aryakia:** software, methodology.

## Data Availability

The data that support the findings of this study are available from the corresponding author upon reasonable request.
